# A Novel Design of an Oral Appliance for Monitoring Electromyograms of the Genioglossus Muscle in Obstructive Sleep Apnea Syndrome

**DOI:** 10.3390/life14080952

**Published:** 2024-07-29

**Authors:** Thamer Y. Marghalani, Ruwaa M. Salamah, Haitham M. Alangari

**Affiliations:** 1 Department of Oral and Maxillofacial Prosthodontics, Faculty of Dentistry, King Abdulaziz University, Jeddah 21589, Saudi Arabia; rdsalamah@stu.kau.edu.sa; 2 Ministry of Health, Madinah 42351, Saudi Arabia; 3 Independent Researcher, Jeddah 23611, Saudi Arabia; haitham.angari@gmail.com

**Keywords:** obstructive sleep apnea, genioglossus muscle, electromyogram, oral appliance, OSA diagnosis, dental device

## Abstract

Obstructive sleep apnea (OSA) is a prevalent source of sleep-disordered breathing. OSA is most commonly associated with dysfunctions in the genioglossus (GG) muscle. In this study, we present the first version of a medical device that produces an electromyogram (EMG) of the GG. The prototype is composed of a (custom-made) 3D-printed mouthpiece. Impressions were taken for the lower arch and scanned with a lab scanner to be converted into digital impressions. ExoCad software was used to design the appliance. Fusion 360 software was then used to modify the design and create tubes to house the electrodes in a bilateral configuration to secure excellent and continuous contact with the GG muscle. Silver–silver chloride electrodes were incorporated within the appliance through the created tubes to produce a muscle EMG. In this preliminary prototype, an EMG amplifier was placed outside the mouth, and isolated electric wires were connected to the amplifier input. To test the design, we ran experiments to acquire EMG signals from a group of OSA patients and a control group in wakefulness. The GG EMGs were acquired from the participants for 60 s in a resting state whereby they rested their tongues without performing any movement. Then, the subjects pushed their tongues against the fontal teeth with steady force while keeping the mouth closed (active state). Several features were extracted from the acquired EMGs, and statistical tests were applied to evaluate the significant differences in these features between the two groups. The results showed that the mean power and standard deviation were higher in the control group than in the OSA group (*p* < 0.01). Regarding the wavelength during the active state, the control group had a significantly longer wavelength than the OSA group (*p* < 0.01). Meanwhile, the mean frequency was higher in the OSA group (*p* < 0.01) at rest. These findings support research that showed that impairment in GG activity continues in the daytime and does not only occur during sleep. Future research should focus on developing the device to be more user-friendly and easily used at home during wakefulness and sleep.

## 1. Introduction

Obstructive sleep apnea (OSA) is the frequent complete or incomplete collapse of the upper airway during sleep, resulting in a repetitive decrease in oxygen saturation for more than 10 s, elevated respiratory effort, fragmented sleep, nocturnal arousal, and daytime sleepiness [1]. The polysomnogram (PSG) is the gold standard procedure for diagnoses and follow-up OSA. It is a complex procedure performed by a sleep technician in a highly specialized and equipped sleep laboratory [2]. An alternative to PSG is the Home Sleep Apnea Testing (HSAT) device, which is patient-friendly, has lower costs, and provides easier access to care [3]. However, the HSAT device does not produce an electroencephalogram (EEG) or measure arousal; thus, OSA may be missed or underestimated. Also, it does not record the Rapid Eye Movement (REM) stage of sleep, and thus, REM-predominant OSA is missed. Senior citizens may find it difficult to fix the sensors, which may dislodge during sleep, resulting in erroneous measurements [4,5]. As a result, diagnostic tools in questionnaires have been developed. Their sensitivity is relatively high, but it is not enough to rule out the possibility of OSA. On the contrary, their specificity is low, resulting in more false-positive diagnoses and limiting the application of these tools in clinical practice [6,7,8].

The genioglossus (GG), an upper airway dilator muscle, consistently demonstrates phasic electromyogram (EMG) activity during inspiration [9]. During sleep, in healthy subjects, there is a rise in upper airway resistance. At sleep onset, GG activity demonstrates a bigger drop in OSA patients than in healthy subjects [10,11]. In OSA and during airway occlusion, GG activity is weakened and becomes less phasic [12,13]. At the end of the apnea epoch, the recovery from airway occlusion is associated with a sudden burst on the GG EMG [12,14]. Interestingly, the genioglossus was shown to have augmented EMG activity in obstructive sleep apnea (OSA) patients compared to healthy cases during wakefulness [15,16]. From a clinical perspective, the accurate analysis of GG fatigue using EMGs is critical to explore the hypothesis that GG fatigue contributes to the pathophysiology of obstructive sleep apnea. 

In this work, we present a novel design of dental appliances that provides suitable and continuous contact between the sensing surface electrodes and the GG muscle. Few attempts have been made to place electrodes intraorally and produce an EMG of the GG [17,18,19,20]. However, these appliances are designed to be used clinically. They are ready-made but do not ensure the comfortable fit and intimate contact needed for the electrodes attached to the GG muscle to function correctly. The literature lacks a reliable and effective method for placing and maintaining surface electrodes in close contact with the GG muscle to record EMG intraorally. A prototype of a preliminary appliance design was tested to acquire a GG EMG from an OSA group and a control group in wakefulness, and the results were compared with what was previously presented in the literature. The project’s ultimate goal will be to have the whole EMG amplifier embedded in the dental appliance so that the device can be used during sleep for a full night of GG EMG monitoring. 

## 2. Materials and Methods

### 2.1. Intra-Oral Appliance Fabrication

For digital fabrication and designing of the Intra-oral appliance, primary impressions using alginate impression materials were taken for the lower arch of the participants. At the same time, the tongue was relaxed to capture the floor of the mouth in the resting position. After that, the diagnostic casts were scanned using a Lab scanner (KAVO ARTICA auto-scan) (KaVo Dental Technologies LLC, Charlotte, NC, USA), and the scanned casts’ stereolithography (STL) files were imported into the ExoCad software. (v. 3.1) Multiple initial designs for the appliance were designed, printed, and piloted by the authors to determine the most comfortable and effective appliance. The optimization criteria are summarized in Table 1.

The approved design of the appliance was created with the Bite-splint Module in ExoCad, and it was produced as follows:

1. The appliance was extended to half the occluso-gingival length of the teeth, except in the lingual anterior area, where the electrode would be placed.
2. The lingual extension was extended to the full depth to the floor of the mouth while the tongue was in the resting position.
3. The width was 1 mm all around, except in the electrode area, where it was 2 mm.

Then, the STL file of the appliance’s primary design was exported to MeshMixer software (v. 3.5.474), where the width of the lingual area was thickened to 2 mm to accommodate the electrodes’ tube placement. After that, the design was exported to Autodesk^®^ Fusion 360^®^ software (v. 16.0.0.1902) to design the tubes used for placing and including the electrodes. Autodesk^®^ Fusion 360^®^ is a cloud-based 3D modeling software platform for producing, designing, and editing, utilizing pre-existing features or model fixtures with integrated CAD\CAM software tools.

**Figure 1:**
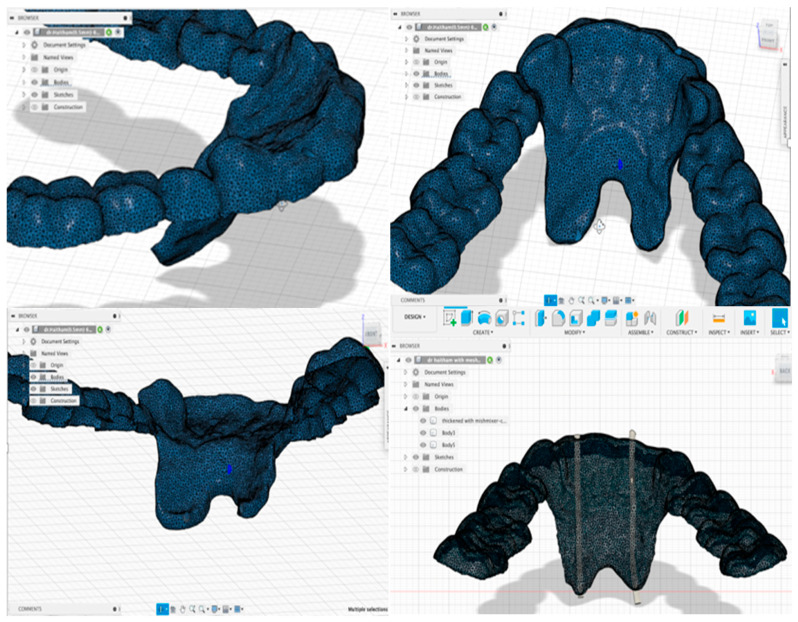
Three-dimensional representation of a finished design ready for 3D printing.

The oral appliance was designed with bilateral electrode configurations for GG EMGs. An electrode tube was created on either side of the mouth with a 1 mm diameter in the area of the lateral incisors and extended from the incisal tip of the tooth to the floor of the mouth with a minimum 10 mm inter-electrode distance. The electrodes were referenced to a common ground lead and placed on the cheek to yield a bipolar recording. The design was 3D-printed using a NextDent^®^ 5100 3D Printer with OrthoFlex material (NextDent, Soesterberg, The Netherlands). The printed appliance was then cleaned according to the manufacturer’s instructions and light-cured with a NextDent LC-3D Printer for 30 min for final polymerization. Figure 1 demonstrates the finished 3D design.

### 2.2. Optimum Device Design

Three aspects of the design were piloted by three of the authors of this study, and the results of each aspect were as follows:

Material thickness: For a 2 mm thick appliance, the material flexibility vanished, and it was uncomfortable to wear, but this was the minimum thickness to protect the electrodes and protect the participants. Therefore, the appliance was printed at a 1 mm thickness, but the thickness was increased to 2 mm in the electrode areas only.

Peripheral extension: The appliance was printed to cover the teeth all around and extend to the lingual sulcus’s depth. This amount of extension was annoying to the patient. Therefore, the material covered only the occlusal 1/3 of the tooth and only had full extension in the electrode area.

Lingual extension: The appliance’s lingual flanges were extended to the full depth of the lingual sulcus in the preliminary design. However, this extension was uncomfortable or even irritating to the participant. Therefore, the extension was reduced to cover the functional depth of the genioglossus muscle.

### 2.3. EMG Amplifier Design

**Figure 2:**
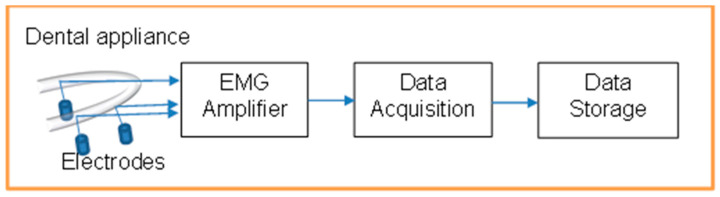
Representation of EMG acquisition process.

The EMG Amplifier was powered by a 3V lithium battery with a micropower instrumentation amplifier (INT122) from Texas Instruments. Silver–silver chloride electrodes were inserted through the mouthpiece to touch the surface of the genioglossus muscles. In this preliminary version, the amplifier was placed outside the mouth and was connected to the sensing electrodes through electrically isolated wires. The EMG activity data were acquired with a data acquisition device from National Instruments (USB-6009-NI) (Austin, TX, USA), and the data were stored on a laptop for further analysis using MATLAB (R2023a) software. Figure 2 shows the process of signal transmission. 

### 2.4. Subject Recruitments and Data Acquisition

To test the comfort of the mouthpiece and stability of the acquired EMG signal, a preliminary study was conducted to compare a group of OSA patients and a control group during wakefulness. This observational study was conducted at King Abdul-Aziz University. The OSA group consisted of eight severe OSA patients who were already diagnosed with OSA based on a diagnosis provided by a one-night PSG in the Center of Sleep Medicine at King Abdulaziz University. The control group consisted of nine subjects who did not have sleep complaints and did not screen positive for risk of OSA based on the Berlin questionnaire [7].

The appliance was checked intra-orally to ensure its correct positioning and comfort. The electrodes were then inserted into their corresponding positions and attached to the panel. The subjects were instructed to keep their tongues at rest while the signal was acquired for one minute. This defines the resting state. Afterward, the participants were instructed to push the tongue against the frontal teeth for 10 s with steady force while keeping the mouth closed. This defines the active state.

### 2.5. Signal Processing and Statistical Analysis

The acquired GG EMG signals were processed offline using MATLAB^®^. Each EMG recording was divided into segments of 5 s in length, and from each segment, seven signal processing features were computed. These features were the mean power, standard deviation, wavelength, mean frequency, zero crossing, slope change account, and sample entropy of the GG EMG. The mean values of the features in the 5 s segments were then determined. Due to the small sample size, a Mann–Whitney U test was performed to test the difference between the EMG features in OSA and the control group in the resting and active states.

### 2.6. Ethical Considerations:

Ethical approval was obtained from the Research Ethics Committee at the College of Dentistry at KAU (approval number: 43107153). Before starting the experiments, the participants signed informed consent forms.

## 3. Results

### 3.1. Sample Size and Participants’ Characteristics

The demographics of the participants in the study are shown in Table 2. 

### 3.2. EMG Results

**Figure 3:**
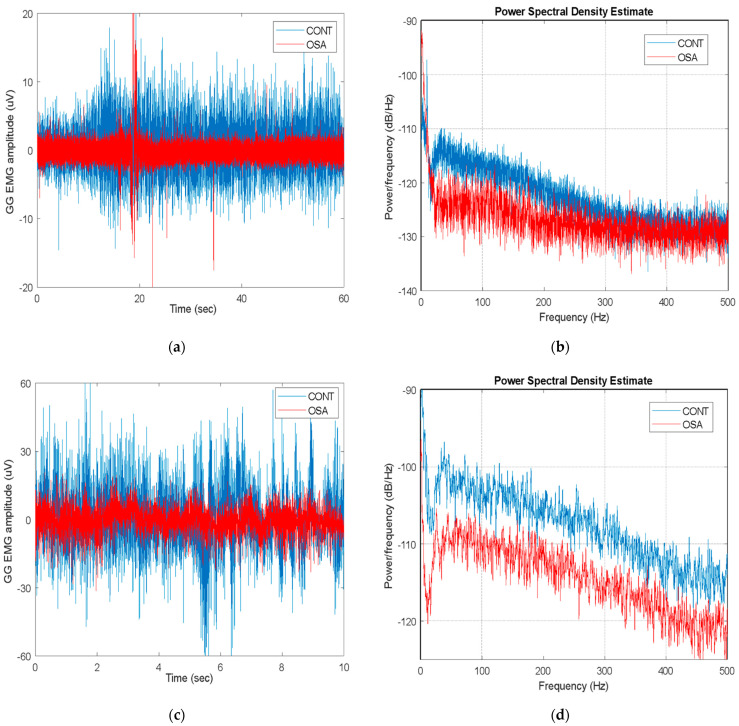
Example of acquired GG EMGs from control and OSA subjects: in resting (top panel) and active states (bottom panel). In both cases, control subject showed higher magnitude of GG EMG than OSA patient, as observed on time (**a**,**c**) and frequency spectra (**b**,**d**).

The magnitude of the GG EMG signals was in the range of a few microvolts, and their frequency bandwidth extended to 450–500 Hz. Both the magnitude and bandwidth increased as the tongue was activated, as shown in Figure 3. 

**Figure 4:**
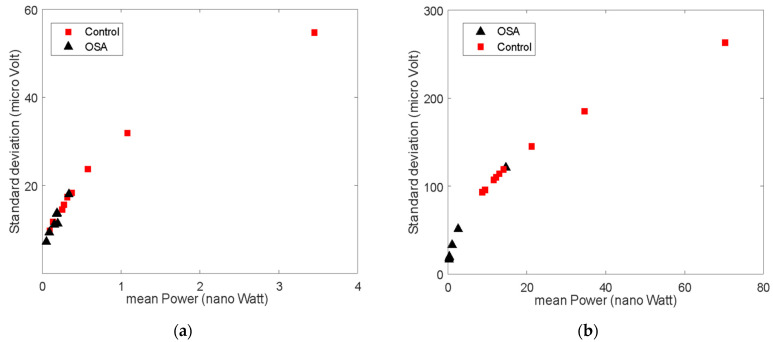
Mean power and standard deviation for EMG signal of control vs. OSA in resting (**a**) and active states (**b**).

The values of GG EMG activity showed differences between the control subjects and the OSA patients (Table 3). The mean power was higher (although not statistically significant) in the control group compared to the OSA group at rest (*p* = 0.055). The standard deviation, however, was statistically significant between the two groups at rest (*p* < 0.05, Figure 4a). 

The mean frequency at rest was significantly higher in the OSA group (*p* < 0.01). In the active state, the power, standard deviation, and wavelength were significantly higher in the control group (*p* < 0.01, Figure 4b). Although no significant differences were found in the mean frequency between the groups, the mean frequency significantly increased for the control group from the resting to the active state (*p* < 0.05). This was not the case for the OSA group. No significant differences were seen in the entropy, zero crossing, or slope change between the two groups in any state. 

## 4. Discussion

In this work, we designed and tested a prototype of a novel dental appliance that allows the acquisition of GG EMGs during wakefulness. The preliminary pilot design consisted of a 2 mm thick appliance that wrapped all of the teeth and extended 2 mm apical to the gingival margin from the buccal/labial side and up to the floor of the mouth from the lingual side. Piloting this design resulted in extreme discomfort and interfered with tongue movement. In addition, the 2 mm thick appliance lacked flexibility. Previous reports have stated that participants preferred custom-made flexible splints and appliances over rigid, ready-made ones due to their resilience and ease of adjustment to the oral anatomy [21,22]. In addition, these studies also reported that participants preferred thin appliances as they interfered less with oral functions [23]. Moreover, the manufacturer’s recommendation for the material was to keep a maximum thickness of 1 mm to provide maximum flexibility and resiliency such that it does not jeopardize the mechanical properties [24].

Therefore, the design was adjusted, and its extension was reduced to cover only the occlusal third of the teeth. The lingual extension extended the full depth to the floor of the mouth to be as close as possible to the GG muscle. The thickness was reduced around the appliance to 1 mm thick, except in the areas of the electrodes and chip, where it was 2 mm to provide appropriate housing for the electrical parts. This design was also piloted, and the participants reported much more acceptance.

Regarding the study results, the strength of the GG EMG was higher for the control group in both the resting and active states. This contradicts what was previously reported in the literature between the OSA and control groups in wakefulness [15,16]. A reason for this could be that our representation of the signal power is an absolute value, while previously, it was presented as a percentage of the maximum EMG value (the max EMG value is usually determined during tongue protrusion). The rise in the EMG power in the active state in our study (which is analogous to protrusion) is much higher in the control group. This makes the ratio of resting power over active power higher in the OSA group. The mean frequency of the control group was significantly lower in the resting state, and it significantly increased for this group from the resting to the active state. This is analogous to the shift in the power spectrum when more skeletal muscle fibers are recruited to raise muscular power [25,26]. This was not observed in the OSA group, which could be due to the fatigue that these muscles experience [27,28]. The variability in the GG EMGs (assessed by the standard deviation and wavelength features) was higher in the control group. This is also related to this group’s higher strength of EMG signal.

The generic post hoc power test for this study will yield a power analysis related to conventional or medium effect sizes to the Mann–Whitney U test. As an example, the calculated effect size (1.29), based on the mean and standard deviation for the mean power feature, yielded a power of 0.67. However, since the data were non-parametric, the Mann–Whitney U test was used, and it is generally robust for small sample sizes [29,30]. Since this is the first study of its kind regarding surface EMG, the next studies will utilize our results to derive their sample size calculations through effect size calculations.

It is also important to note that the BMI and age do not match between the OSA and the control groups, which might have affected the results. The control group did not undergo PSG diagnosis. We performed screening using the Berlin test, which is sensitive for detecting OSA [8]. Again, this is a preliminary study, and the same patients or other groups may undergo PSG in a further series of studies that might confirm these results and findings. In addition, the tests were conducted only during wakefulness. These findings, however, encourage further investigations that include new wearable versions of the amplifier, which can be tested at home for acquiring GG EMGs on different days and at multiple times during the day in wakefulness. A comparison between GG EMGs and polysomnography (the gold standard) during sleep will be considered with the amplifier fully embedded in the mouthpiece, which is the ultimate goal of our research work.

## 5. Conclusions

In conclusion, this study aims to develop an oral appliance that can be used at home to place surface electrodes in close contact with the GG muscle during sleep to record EMG signals for the purpose of the detection (or screening) of OSA.

Multiple prototypes were developed and tested; the best design in terms of results and comfortability was the one that covered half the occluso-gingival length of the teeth buccally and extended up to the floor of the mouth lingually. The most suitable material thickness was 1 mm all over, except over the electrode areas. OrthoFlex (NextDent) resinous material was used to fabricate the appliance. It has suitable strength, resiliency, and flexibility to house the electrical components and is comfortable in the patient’s mouth.

EMG features are numerous, and multiple features were recorded in this clinical trial. The mean power, standard deviation, and wavelength were significantly higher in the control group compared to the OSA group during the active state. Also, monitoring changes in the GG EMG spectrum might help in understanding the effect of fatigue on the GG muscle. However, these results were recorded during wakefulness, and the results may differ during sleep.

Future research should focus on embedding the miniaturized chip and electrodes within the device to form a single, easy-to-use appliance for home use with a larger sample size and testing the device during sleep.

## Figures and Tables

**Table 1:** Optimization criteria for appliance design.

	Design 1	Design 2
Material thickness	2 mm thickness all around	2 mm thickness in the electrode areas only
Peripheral extension	Full extension around teeth and lingual area	Full extension at the electrode sites only with extension to 1/3 the occlusal surface of the teeth
Lingual extension	The flange was extended to the full depth	The flange was extended to the functional depth only

**Table 2:** Demographic information of the study participants.

OSA Patients					
Subject	Age	Gender	BMI *	AHI †	Use of CPAP ‡
O1	81	M	35.9	36.2	No
O2	77	M	29.4	33.6	No
O3	51	F	44.6	33.5	Yes
O4	83	M	26.6	47.5	n/a
O5	48	M	35.5	41.4	No
O6	35	M	29.6	33	n/a
O7	49	M	39.5	74.1	No
O8	62	M	30.8	51.9	No
**Median (Q1, Q3)**	56.5 (48.8, 78)	--	33.2 (29.6, 36.8)	38.8 (33.6, 48.6)	--
**Control Group**					
C1	49	M	30.5	n/a	No
C2	32	M	32.7	n/a	No
C3	74	M	28.8	n/a	No
C4	29	F	25.3	n/a	No
C5	28	F	23.3	n/a	No
C6	28	F	21.6	n/a	No
C7	30	M	31.9	n/a	No
C8	27	M	23.3	n/a	No
C9	24	M	21.7	n/a	No
**Median (Q1, Q3)**	29 (28, 32)	--	25.3 (23.3, 30.5)	--	--

* BMI: Body Mass Index, † AHI: Apnea-Hypopnea Index, ‡ CPAP: Continuous Positive Airway Pressure.

**Table 3:** EMG features in resting and active states for both OSA and control groups.

Feature	Resting State		Active State	
	OSA Median (Q1, Q3)	Control Median (Q1, Q3)	OSA Median (Q1, Q3)	Control Median (Q1, Q3)
Mean power (nanoWatt)	0.18 (0.11, 0.19)	0.32 (0.25, 0.58)	0.4 (0.35, 1.85)	13.1 (11.6, 21.2) *
Standard deviation (μV)	11.3 (9.7, 13.6)	17.3 (14.5, 23.8) **	19.7 (18.6, 41.9)	114.2 (107.5, 145) *
Wavelength (mV)	6.9 (5.6, 8.3)	7.51 (7.47, 14.7)	12.7 (11.7, 26.7)	81.6 (69.3, 102.6) *
Mean frequency (Hz)	107.7 (102.9, 111.6)	91.5 (82.1, 93.1) *	110.2 (75.5, 118.5)	113.9 (96.3, 126.7)
Zero crossing	284.0 (266.9, 296.4)	265.8 (237.1, 280.9)	273 (256, 297.5)	295.5 (255.5, 315.5)
Slope change	395.8 (376.1, 404.0)	388.3 (376.8, 402.6)	390.5 (366, 406.5)	410.5 (371.5, 418.5)
Sample entropy	1.88 (1.70, 1.99)	1.83 (1.64, 1.95)	1.71 (1.43, 1.83)	1.71 (1.41, 1.74)

** Control group is significantly different from OSA, with *p* < 0.05; * control group is significantly different from OSA, with *p* < 0.01.

## Data Availability

Data available on request due to restrictions to privacy.
